# Breast tuberculosis cases rising in Sicily

**DOI:** 10.1016/j.ijscr.2018.09.048

**Published:** 2018-10-08

**Authors:** Angela Strazzanti, Claudio Trovato, Santi Gangi, Francesco Basile

**Affiliations:** Departement of General Surgery, Senology University Hospital of Catania, Catania, Italy

**Keywords:** FNA, fine needle aspiration, TM, mammary tuberculosis, TB, breast tuberculosis, TM, mammary tuberculosis, GM, Granulomatous mastitis, Case reports, Tuberculosis, Extra-pulmonary localization, Granulomatous mastitis, Scrofola, Fine nipple aspiration

## Abstract

**Introduction:**

Our work has been reported in line with the SCARE criteria [21].

Mammary tuberculosis (TM) is an extremely rare condition (Khanna et al., 2000 [1]). It has been estimated to be 0.1% of breast lesions examined histologically, but it reaches 3–4% in countries such as India and Africa where the disease shows a high incidence. This disease can present a diagnostic problem in radiological and microbiological investigations, and thus extreme caution is necessary.

**Presentation of case:**

A 26 year-old Eritrean female with a personal history of HIV infection came to our Emergency Department showing generalized limphoadenopathy and weakness in addition to a huge right breast mass. Our examination revealed a tender mass measuring 12 × 10 cm that involved all right breast. Her skin was not erythematous and local temperature was normal. She was painless.

Fine needle aspiration (FNA) showed a milky and greenish shaded fluid.

**Conclusions:**

Nowadays the differential diagnosis between breast cancer and breast tuberculosis is very important, mostly in Italy and in particular in Sicily, where the massive influx of immigrants has compelled physicians to reconsider the presence of !tuberculosis.

## Highlights

• Mammary tuberculosis (TM) is an extremely rare condition.
• The differential diagnosis between breast cancer and breast tuberculosis is very important.
• It was possible to identify the *Mycobacterium tuberculosis* in between nucleic acid probes and PCR.

## 1 Introduction

Mammary (breast) tuberculosis is a rare manifestation of extra-pulmonary localization of the disease which accounts for less than 0.1% of breast conditions in developed countries [1]. Breast tuberculosis (TB) was first described by Sir Astley Cooper in 1829 [2]. It mostly appears in women of reproductive age, multiparous, lactating. It has been scarcely reported to infect male patients, mainly before puberty, as well as women of older age.

The incidence of isolated TB of the breast in the world ranges from 0.10% to 0.52% and is scarcely reported even in countries with a high incidence of tuberculosis infection [6]. It is explained by a noticeable resistance of the mammary tissue to the *Mycobacterium tuberculosis* [7].

Hani-Bani et al. [4] believed that immigration from endemic areas, and the increasing prevalence of immunosuppressive disorders, including HIV infection, and the development of drug resistant strains of *Mycobacterium tuberculosis*, might be responsible for a future increasing incidence of TM in Western Countries.

Moreover, the disease is not easily diagnosed because of its physical similarity to carcinoma, bacterial abscesses and other granulomatous diseases like idiopathic Granulomatous Mastitis (GM, an uncommon breast lesion that was first described by Kessler and Woolloch [5]) which are all on the increase.

## 2 Clinical case

**Fig. 1:**
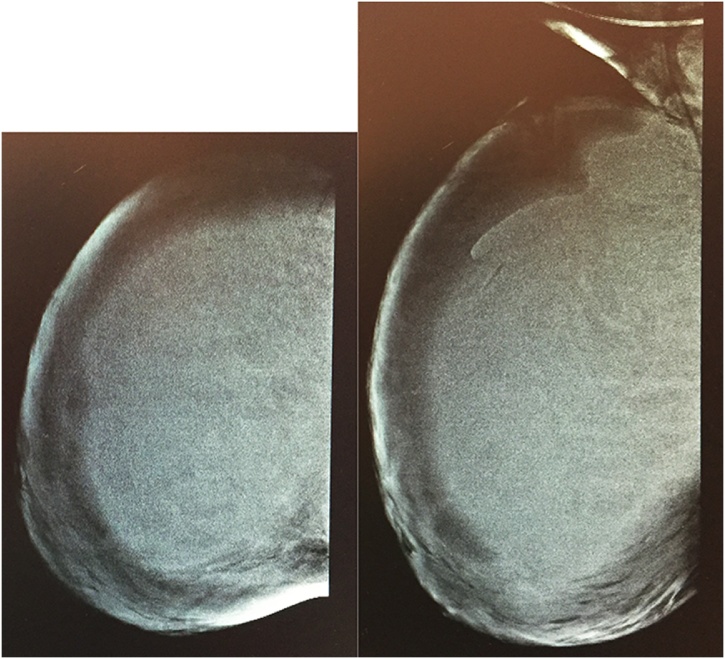
Mammography CC and LL dx.

We report a case of breast tuberculosis that was treated in the Breast Unit of our hospital. A 26 year-old Eritrean female with a personal history of HIV infection and familiar history of breast cancer (her sister had died at the age of 34) came to our Emergency Department showing generalized limphoadenopathy and weakness in addition to a huge right breast mass. The patient was subjected to a CT scan of neck, abdomen and chest, that revealed a big mass in the right breast of about 10 × 7 cm, bilateral latercervical colliquated lymph nodes, the biggest being 27 × 20 mm, many right ascellary and retropectoal lymph nodes, and paraaortic, celiac and paracavale lymph nodes, but no nodes in the chest and no pleural effusion. For this reason she was hospitalized in Medicine Department with a suspicious diagnosis of lymfoma. During the stay, she underwent a mammography (Fig. 1) which showed a generalized increased radioopacity spread throughout the right breast.

Ultrasound scan was apparently useful for characterising the ill-defined breast mass and excluding the presence of solid masses. But the findings of a hypoechoic lesion with heterogeneous internal echoes and irregular borders were not specific ones. The Color Doppler showed increased circumferential vascularization in avascular centered lesions which might be interpreted as a sign of continuity of the infective process. For this reason it was diagnosed a breast abscess [14].

While being treated with antinflammatory drugs and a aspecific antibiotica therapy, the patient came up to our Breast Surgery Unit Department because she had not had any improvement of symptoms.

Our examination revealed a tender mass measuring 12 × 10 cm that involved all right breast. Her skin was not erythematous and local temperature was normal. She was painless. The mass was soft in consistency, floating and mobile on the chest wall and there were no signs of inflammation. It meant the possible presence of a “cold abscess” or a big cystis. Moreover, there was a clinically palpable right axillary lymphadenopathy.

The total leukocyte count and erythrocyte sedimentation rate were low; the other blood tests were normal except for the electrophoretic protidogram where we found a high level of gamma globuline.

Fine needle aspiration (FNA) showed a milky and greenish shaded fluid. On needle aspiration, some thick pus came out and it was drained out of about 200 ml. The pus was sent for bacteriological, citological and coltural test, the results of which were negative for aerobic, anaerobic bacteria and fungi.

The patient had a latercervical lymph node core biopsy that exluded lymphoma. The evaluation of the specimen revealed only inflammatory cells, including neutrophils, lymphocytes, and abundant macrophages, granulomas with central caseaous necrosis and epitheloid histiocytes. But by means of nucleic acid probes and PCR, it was possibile to identify the *Mycobacterium tuberculosis* [20]. The laterocervical limph node was due to a scrofulous swelling.

The patient was put on anti-tubercular drugs treatment (rifampicin 600 mg, isoniazid 300 mg, pyrazinamide 1500 mg and ethambutol 1000 mg per day) for 2 months which continued with the addition of rifampicin and isoniazid therapy for 4 additional months. In the follow-up period of 6 months, the patient recovered very well and was advised to continue the treatment.

At the end of the antituberculous treatment period, the patient appeared to be clinically and radiologically without any evidence of residual disease.

## 3 Discussion

Mammary tuberculosis is an extremely uncommon disease entity, especially among European populations, because organs or tissues like the breast, skeletal muscle and spleen are more resistant to infection, making the survival and multiplication of the tubercle bacilli difficult [8,9]. It should be considered in immunodeficiency states like HIV infection [10].

In the European countries where it is detected it represents less than 0.1% of the mammary lesions examined via histology [4,7].

The incidence of isolated TB of the breast remains low, ranging from 0.10% to 0.52%. In the high tubercular endemic countries like Africa, the incidence varies from 0,1% to 3.59% including lactating females. The advent of multidrug resistant strains and HIV infection is steadily increasing its extra-pulmonary manifestation in developing countries and predominantly among migrant population. (*Soliman MS, Lessnau k, Hasmat A (2007*)).

The highest tubercular endemic European countries are Portugal, Malta, Estonia where tuberculosis is associated with HIV infection, but we do not have any specific data about breast tuberculosis [11].

We have not been able to find data as regards incidence in Italy, but we know that in 2016 there were 4032 cases of TB notification [11].

TB of the breast usually affects women aged between 20 and 50 years [3,6]. Lactating women appear to be at higher risk, probably due to the increased blood supply in the breast and to the dilatated ducts, making them more vulnerable to lacerations and infections [12].

Although mammary tuberculosis is much more common in females, it has been previously reported to also occur in males. Tuberculosis mastitis is usually unilateral, it seldom affects male patients and should be considered in immunodeficiency states like HIV infection.

The commonest location of the lump in the breast is the central or upper outer quadrant of the breast [13]. The mass may be fluctuant and is usually covered with indurated tissue.

Breast tuberculosis may be classified into three types, namely: nodular, disseminated and sclerosing varieties. McKeown and Wilkinson classified tuberculosis of the breast into five different types [15]: (A) nodular tubercular mastitis, (B) disseminated or confluent tubercular mastitis, (C) sclerosing tubercular mastitis, (D) tuberculous mastitis obliterans, and (E) acute miliary tubercular mastitis.

They classified breast tuberculosis as primary when the breast lesion was the only manifestation of tuberculosis, and secondary when there was a demonstrable focus of tuberculosis elsewhere in the body.

As regards our case, we think it is an example of secondary mammary tuberculosis, because another tuberculous focus was found in laterocervical limph nodes. Involvement of the breast in such cases of secondary tuberculous infection is presumed to be the result of direct hematogenous spread.

The first case of mammary tuberculosis was recorded by Sir Astley Cooper in 1829 who called it “scrofulous swelling of the bosom”.

The nodular form is the most common variety and is characterized by a well defined, painless, slow growing caseous lesion in the breast. Involvement of overlying tissue is usually late and it is at this point that the mass becomes painful. As in our case, the patient presented breast cold abscess, without any inflammation elements and, on cytological diagnosis, it came as tuberculosis, which is very rare.

The common mammographic findings are coarse stromal texture with or without an ill-defined breast mass and skin thickening, which are all nonspecific for a diagnosis [15]. Sclerosing tubercular mastitis reveals a homogenous dense mass with fibrous septa and nipple retraction.

Lesions due to tuberculosis have no specific ultrasonographic findings. They are observed as heterogeneous, hypoechoic, irregular bordered masses with internal echoes, or sometimes as thick-walled cystic lesions that show internal septa and posterior acoustic enhancement [16].

Ultrasound helps evaluate if the chest wall is affected by the lesion. The mammographic and sonographic features of tubercular mastitis in a study by Sakar and group found mass lesion mimicking malignant tumors (30%), smooth bordered masses (40%), axillary or intramammary adenopathy (40%), asymmetric density and duct ectasia (30%), skin thickening and nipple retraction, macrocalcification (20% each), and skin sinus (10%). On ultrasound, 60% had hypoechoic masses, 40% focal or sectorial duct ectasia, and 50% axillary adenopathy [17].

Color Doppler US findings of breast tuberculosis are not mentioned in the literature.

FNA is generally a reliable diagnostic procedure, particularly if the aspirated material can be examined by the stains for acid fast bacilli [18,19]. In our present report, FNA was performed but not diagnostically useful for mammary tuberculosis. The accurate diagnosis was only achieved after cytological evaluation of the laterocervical lymph node.

## 4 Conclusion

We can conclude that nowadays the differential diagnosis between breast cancer and breast tuberculosis is very important in our country, where the massive influx of immigrants has obliged physicians to reconsider the presence of tuberculosis.

Breast tuberculosis represents a rare disease that should always be suspected when evaluating cases of breast abscesses, fistulae or nodules, with poor response to classical non-tuberculosis antibiotic treatment.

Tuberculosis of the breast is uncommon even in countries where the incidence of pulmonary and extrapulmonary tuberculosis is high, however it must be suspected if patients come from India or Africa, where there is the highest incidence of tuberculosis.

Physicians should consider this clinical entity, often mimicking breast cancer, especially in times of migrational surges and in patients with HIV infection.

This disease can highlight a diagnostic problem on radiological and microbiological investigations, and thus a high index of suspicion is needed. Incorporating a highly sensitive technique like PCR may be helpful in establishing the usefulness of such technology and can aid in confirming an early diagnosis. The disease is curable with antitubercular drugs, and surgery is rarely required.

## Conflicts of interest

No conflict.

## Sources of funding

No sources.

## Ethical approval

Approval has been given by the University of Catania ethics committee.

## Consent

Written consent has been given.

The information and images of patients used is essential for scientific purposes and explicit permission has been given as part of the consent.

Identifying characteristics are altered to protect anonymity, we provide assurances that such alterations do not distort scientific meaning.

## Author contributions

Trovato Agata: Literature review and writing the article.

Nicola Pacini MD: Reviewing and editing the article.

## Registration of research studies

N/A.

## Guarantor

Prof. Francesco Basile.

## Provenance and peer review

Not commisioned, externally peer reviewed.
